# Structural Optimization of Foldamer-Dendrimer Conjugates as Multivalent Agents against the Toxic Effects of Amyloid Beta Oligomers

**DOI:** 10.3390/molecules23102523

**Published:** 2018-10-02

**Authors:** Éva Bartus, Gábor Olajos, Ildikó Schuster, Zsolt Bozsó, Mária A. Deli, Szilvia Veszelka, Fruzsina R. Walter, Zsolt Datki, Zsolt Szakonyi, Tamás A. Martinek, Livia Fülöp

**Affiliations:** 1 Department of Medical Chemistry, University of Szeged, Dóm tér 8, H-6720 Szeged, Hungary; bartus@pharm.u-szeged.hu (E.B.); olajosg@pharm.u-szeged.hu (G.O.); schuster.ildiko@med.u-szeged.hu (I.S.); bozso.zsolt@med.u-szeged.hu (Z.B.); martinek@pharm.u-szeged.hu (T.A.M.); 2 Institute of Biophysics, Biological Research Center of HAS, Temesvári krt. 26, H-6726 Szeged, Hungary; deli.maria@brc.mta.hu (M.A.D.); veszelka.szilvia@brc.mta.hu (S.V.); walter.fruzsina@brc.mta.hu (F.R.W.); 3 Department of Psychiatry, University of Szeged, Kálvária sgt. 57, H-6725 Szeged, Hungary; datkiz@yahoo.com; 4 Institute of Pharmaceutical Chemistry, University of Szeged, Eötvös u. 6, H-6720 Szeged, Hungary; szakonyi@pharm.u-szeged.hu

**Keywords:** amyloid β, molecular recognition, foldamer, protein aggregation, multivalency

## Abstract

Alzheimer’s disease is one of the most common chronic neurodegenerative disorders. Despite several in vivo and clinical studies, the cause of the disease is poorly understood. Currently, amyloid β (Aβ) peptide and its tendency to assemble into soluble oligomers are known as a main pathogenic event leading to the interruption of synapses and brain degeneration. Targeting neurotoxic Aβ oligomers can help recognize the disease at an early stage or it can be a potential therapeutic approach. Unnatural β-peptidic foldamers are successfully used against many different protein targets due to their favorable structural and pharmacokinetic properties compared to small molecule or protein-like drug candidates. We have previously reported a tetravalent foldamer-dendrimer conjugate which can selectively bind Aβ oligomers. Taking advantage of multivalency and foldamers, we synthesized different multivalent foldamer-based conjugates to optimize the geometry of the ligand. Isothermal titration calorimetry (ITC) was used to measure binding affinity to Aβ, thereafter 3-(4,5-dimethylthiazol-2-yl)-2,5-diphenyltetrazolium bromide (MTT) based tissue viability assay and impedance-based viability assay on SH-SY5Y cells were applied to monitor Aβ toxicity and protective effects of the compounds. Important factors for high binding affinity were determined and a good correlation was found between influencing the valence and the capability of the conjugates for Aβ binding.

## 1. Introduction

Alzheimer’s disease (AD) is one of the most common forms of senile dementia and it belongs to the aggregation-associated disorders [1,2]. The pathology of AD is not yet fully understood and simultaneous effects of several different factors may be responsible for the onset of the disease. It is supported by numerous pieces of evidence that self-association of the amyloid β (Aβ) peptide into non-native structures plays a key role in AD pathogenesis [3,4]. The mechanism of cytotoxicity and the types of toxic aggregates are still unknown, but the current hypothesis suggests that small soluble aggregates drive the degenerative pathology and their amount in the cerebrospinal fluid shows the best correlation with the loss of memory and cognitive function [5,6,7]. Several potential disease-modifying therapeutic strategies are at preclinical or clinical stages of development, aiming either to prevent the formation of the protein aggregates and/or to eliminate them. These attempts involve the inhibition of the biosynthesis of Aβ peptides by β- and γ-secretase modulators [8,9], the removal of the produced Aβ with catalytic antibodies [10] and the removal of Aβ using passive or active immunization [11,12,13]. Inhibiting the Aβ oligomerization is the most investigated strategy to reduce the level of synaptotoxic forms of the peptide [14,15,16,17].

Targeting aggregated forms can be a potential therapeutic approach for the treatment of these self-association related disorders such as AD, Parkinson’s, and Huntington’s disease [18] but the inhibition of protein-protein interactions (PPIs) is a real challenge [19,20]. Small molecule inhibitors can target such PPIs in which the pharmacophoric anchor points are concentrated in relatively small binding clefts [20,21] but the lack of such a well-defined binding site makes difficult for them to recognize a large (typically 1000–2000 Å^2^), solvent exposed, flat protein surface [22,23]. Although peptides and certain proteins such as monoclonal antibodies are promising drug candidates to modify PPIs by recognizing such molecular targets with high affinity [24,25,26], due to their several disadvantages like the unfavorable pharmacokinetic properties, there is a growing interest for peptidomimetics. The incorporation of unnatural β-amino acids into an α-peptide chain results in a heterogeneous-backbone oligomer and these peptidomimetics, so called foldamers, can mimic structural and functional features of biomolecules [27]. Even relatively short foldamer sequences can attain well-defined secondary structure elements [28,29,30] and several studies demonstrate the efficiency of β- and α/β-peptides in targeting protein-protein interactions [31,32,33,34].

We have reported a tetravalent β-peptidic dendrimer conjugate which can selectively recognize the low molecular weight (LMW) fraction of Aβ (1–42) and binds to peptide assemblies without remodeling its conformation or disaggregating oligomers into monomers with random coil structures. The conjugate possessed a high affinity-binding to Aβ and displayed a protective effect against cytotoxic Aβ in a long-term potentiation (LTP) model on hippocampal slice [35]. In this work, the structural optimization of the multivalent foldamer-dendrimer conjugate was carried out and the structure-activity relationship was analyzed in two aspects: (i) Testing the effect of the side chain chemistry alteration of the β-peptidic recognition unit, (ii) testing the effect of multivalence on Aβ binding and fine tuning the topology of the multivalent interaction.

## 2. Results and Discussion

### 2.1. Effects of Side Chain Alteration on Aβ Binding

**Figure 1:**
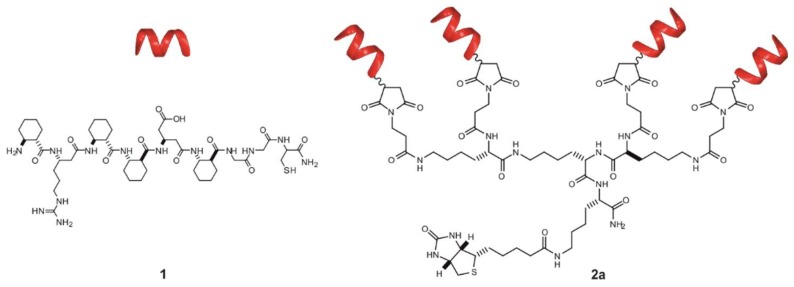
Sequence and schematic representation of recognition unit (**1**) and tetravalent conjugate (**2a**).

We reported previously that the unnatural β-peptide **1** (Figure 1) can create a weak interaction with Aβ and coupling these amyloid recognition segments to a tetravalent-zero generation poly(amidoamine) (PAMAM) dendrimer results in a multivalent biomimetic molecule with selective low nanomolar binding affinity to low molecular weight (LMW) fraction of the Aβ oligomers [35]. The conformation of the short helical recognition unit and the relative position of the ionic residues were key factors for Aβ binding.

**Figure 2:**
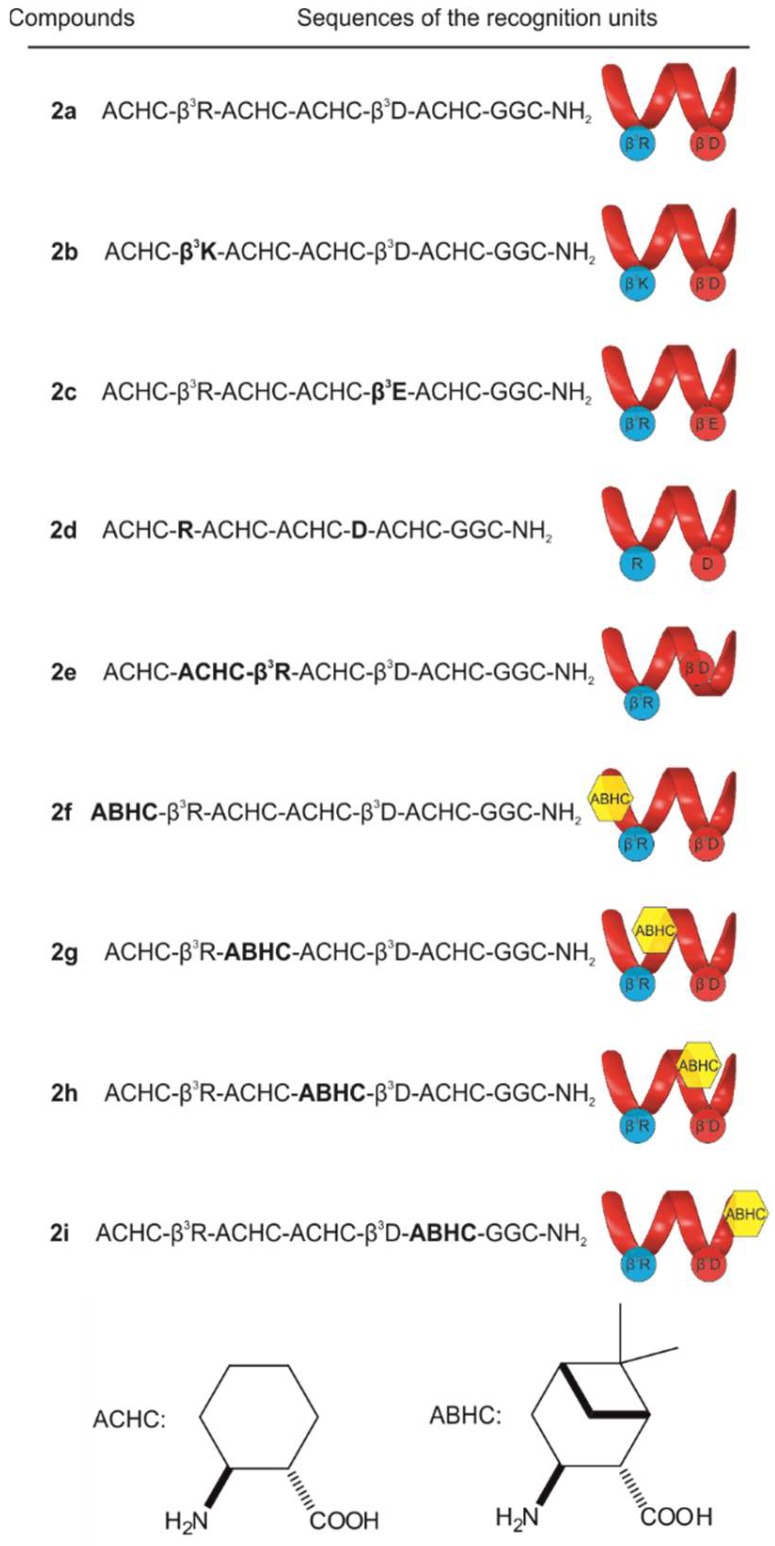
Sequence and schematic structure of the helical recognition segments of conjugates (**2a**–**2i)**. β^3^h preposition indicates the beta(3)-homologue of the corresponding alpha amino acid (hereinafter find in the text as β^3^hXaa), ACHC: (*1S*,*2S*)-aminocyclohexane-carboxylic acid, ABHC: (*1S,2S,3S,5R*)-3-amino-6,6-dimethylbicyclo [3.1.1] heptane-2-carboxylic acid.

To study the role of certain structure elements in the binding affinity, a set of new foldamer sequences were designed (Figure 2) and ligated to lysine-dendron scaffolds having a focal symmetry. This template with biotin affinity tag can be easily synthesized by solid phase peptide synthesis and immobilized on a streptavidin coated support such as the surface of the ELISA plate [36]. Structural changes included fine-tuning the side-chain properties (**2b**–**2d**), changing the position of ionic residues (**2e**) and introducing bulky bicyclic side-chains (**2f**–**2i**).

The binding affinities of **2b**–**2i** to Aβ oligomers were tested in ITC experiments. In such cases where the fitting failed due to low heat response or poor solubility, sandwich ELISA experiments were performed to enable the comparison of all conjugates (see Supporting Information for details). ITC experiments revealed that the initial tetrameric conjugate (**2a**) displayed a two-stage high affinity interaction (K_D1_ = 27.63 ± 7.74 nM) with the Aβ (Table 1, Figure S1).

Although homologous replacements of the charged side-chains modulated the affinity, they could be basically tolerated. The β^3^hArg to β^3^hLys exchange (**2b**) caused a decreased affinity at the first binding stage, whereas β^3^hAsp to β^3^hGlu change (**2c**) led to somewhat improved properties; the β^3^h preposition indicates the beta(3)-homologue of the corresponding alpha amino acid. The effect of the β^3^hArg to β^3^hLys replacement can be explained with the structural differences between the charged side chains. In contrast to Lys, where the charge is localized on the terminal aliphatic amino group, in the case of Arg, the positive charge is delocalized within the π-bonded system of the guanidinium ion, resulting in a considerably different charge distribution and geometry. This may result in distinct physicochemical properties of the peptides associated with a different binding affinity [37]. In case of **2c**, the evidence suggests that, the presence of the negatively charged residue in position 5 is essential for the binding [35], but the length of the side chain did not have an important role. In the ITC experiment of **2d**, in which we replaced β^3^hAsp to Asp, and β^3^hArg to Arg, the observed low ΔH made the fitting difficult. A similar low-heat response was found for **2e**, where we changed the position of the positively charged residue (β^3^hArg) in the helix, and in this case the fitting also failed. A systematic ACHC to ABHC exchange was also carried out along the chain (**2f**–**2i**), and we found that this approach did not lead to a significant increase in the affinity at the first binding stage (Table 1). ACHC to ABHC replacement in the helix considerably decreased the solubility of the conjugates, which made the dissolution difficult in the target concentration, therefore resulted relatively noisy thermographs in case of **2f**, **2g** and **2h**, furthermore the K_D_ of **2i** could not be determined under these conditions.

The apparent dissociation constants for the complete set of new conjugates were calculated individually from the data of the ELISA experiments as well. Comparing the K_D_ values of **2f**–**2i** in this case, the replacement of ACHC to ABHC had only a minor effect on the Aβ binding. The cyclic β-amino acid ACHC not only provides the helical conformation of such a short foldamer but also protects the ionic residues from solvent upon binding due to its hydrophobicity. Increasing the bulkiness of the helix by a substitution of ACHC with a bicyclic amino acid in the recognition element resulted in a retained binding affinity to Aβ. In the experimental setup of ELISA, K_D_ for **2d** and **2e** could also be determined and possessed increased values in both cases compared to **2a** (Table 1, Figure S2). In case of **2d** the results indicated that the simultaneous replacement of β^3^hArg and β^3^hAsp to the α-analogues could be moderately tolerated, which revealed the importance of the conformation of the helical recognition segment. Interestingly, changing the relative position of the charged residues (**2e**), which results in an altered orientation of the ionizable side chains from parallel to antiparallel, did not influence the binding of the molecule to Aβ considerably, thus some flexibility may be assumed regarding the structure of the foldamer. Since none of the analogues could exert significantly improved binding affinities to Aβ, the foldamer **1** was used as a recognition unit in the further studies.

### 2.2. Quantitative Study of the Effects of Multivalency and Topology on Aβ Binding

**Figure 3:**
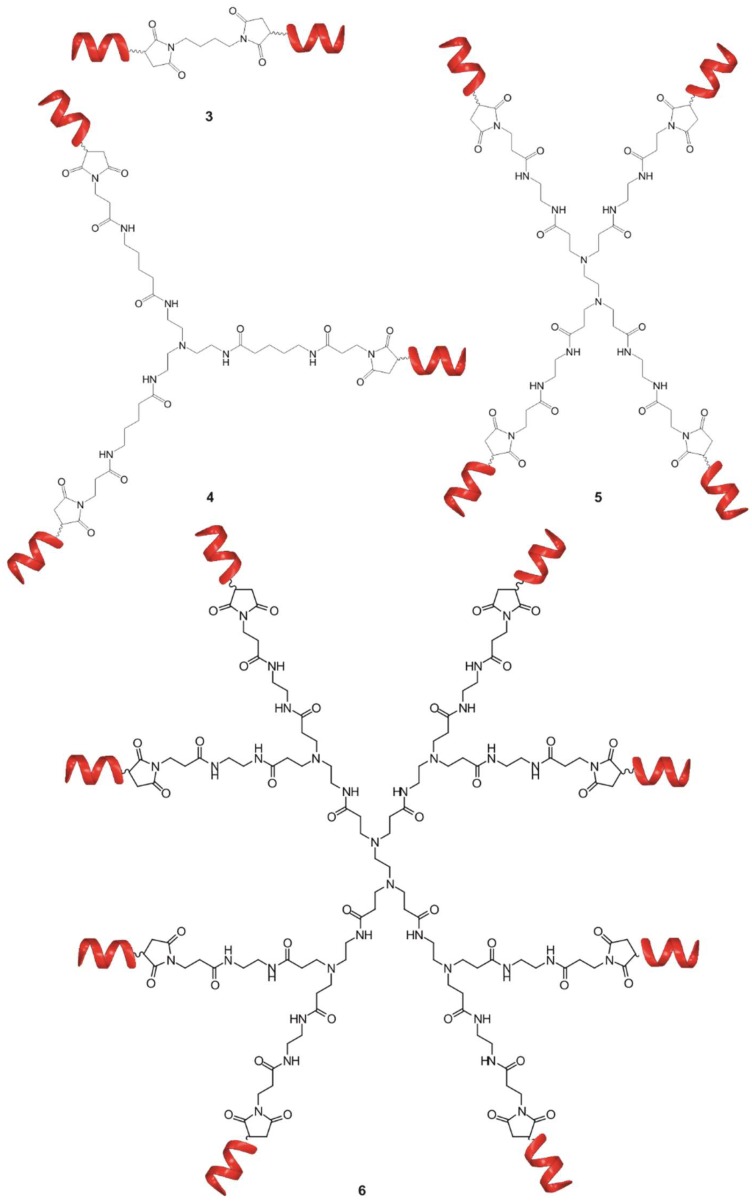
Schematic representation of bivalent (**3**), trivalent (**4**), tetravalent (**5**) and octavalent (**6**) foldameric conjugates.

To examine the effects of multivalency, di-, tri-, tetra- and octa-valent conjugates were synthesized by coupling foldamer helices (**1**) to different scaffolds (Figure 3). ITC was used to determine thermodynamic parameters of the binding of different conjugates.

As we reported previously, the foldameric fragment **1** (Figure 1) and the divalent conjugate **3** (Figure 3) displayed a low micromolar binding affinity. For compounds **4**–**6**, two stage enthalpograms were recorded (Table 2, Figure S3) in the solution phase ITC experiments. In contrast to the divalent conjugate **3**, (K_D_ = 0.721 ± 0.12 µM), the trivalent **4** and tetravalent **5** ones possessed a low nanomolar binding to Aβ and there is a good correlation between the multivalence and binding constants (Table 2). Increasing the number of arms from four to eight did not further improve the affinity in case of **6**, (K_D1_ = 69.0 ± 12.0 nM) thus, the tetravalent form was selected for fine tuning the topology of the conjugate.

**Figure 4:**
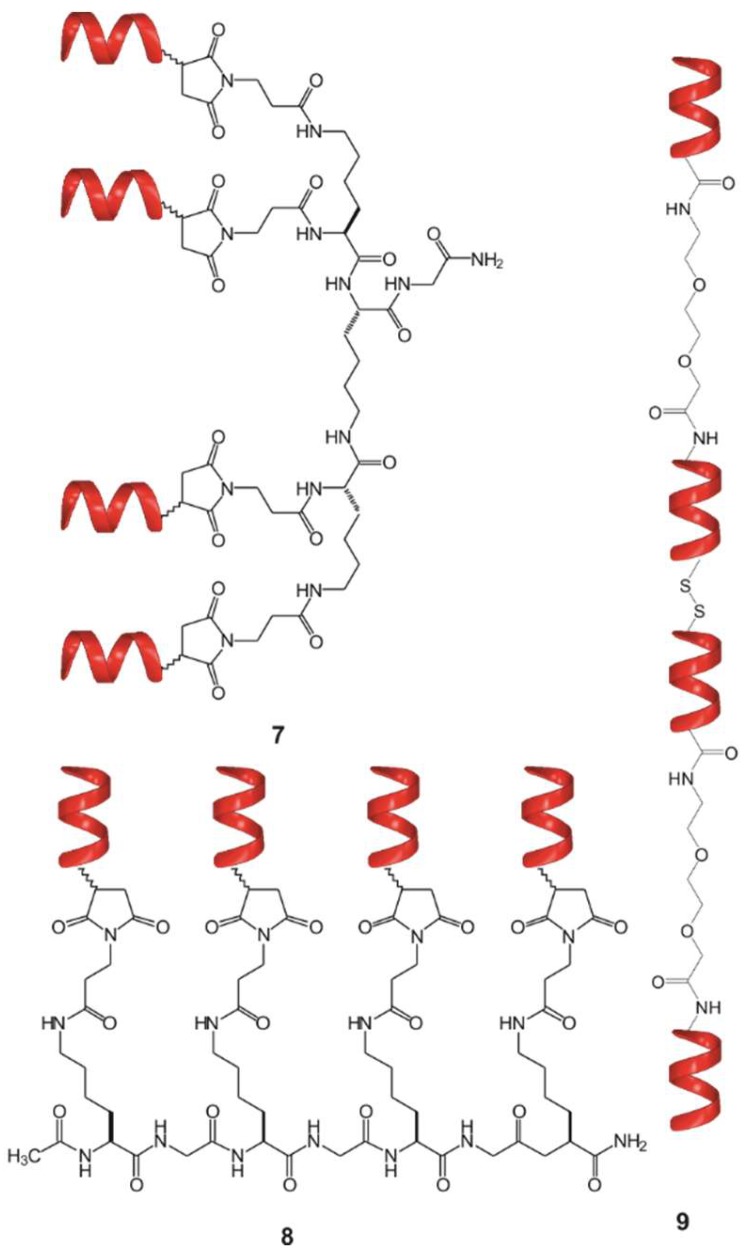
Tetrameric foldamer conjugates with different topology of interaction: focal symmetry (**7**), linear, foldamers are connected in parallel (**8**), linear tetramer (**9**).

Following two different coupling strategies, beside the centrally symmetric **5** and focally symmetric **7**—which is equivalent with **2a**, without the affinity tag—, two other tetravalent foldamer- conjugates were synthesized: (i) The short foldamer sequences were attached in parallel to a linear template (**8**), (ii) the helical recognition segments were coupled in a sequential manner into a linear polymer (**9**) (Figure 4).

ITC was used to test the binding of the different conjugates with different topologies to Aβ (**7**–**9**, Table 2). Based on the results, the linear tetramer **9** has a slightly decreased binding affinity to Aβ. The possible reasons for this phenomenon could be the loss of two of four free N-terminal amino groups of the foldamers and the decrease of mobility of the recognition units in this geometry. In the cases of the branched tetravalent conjugates (**5**, **7** and **8**), low nanomolar K_D_ values were found belonging to the first binding stage (Table 2, Figure S3), probably owing to the lack of steric hindrance mentioned above.

### 2.3. Effects of Multivalency on Biological Activity

**Figure 5:**
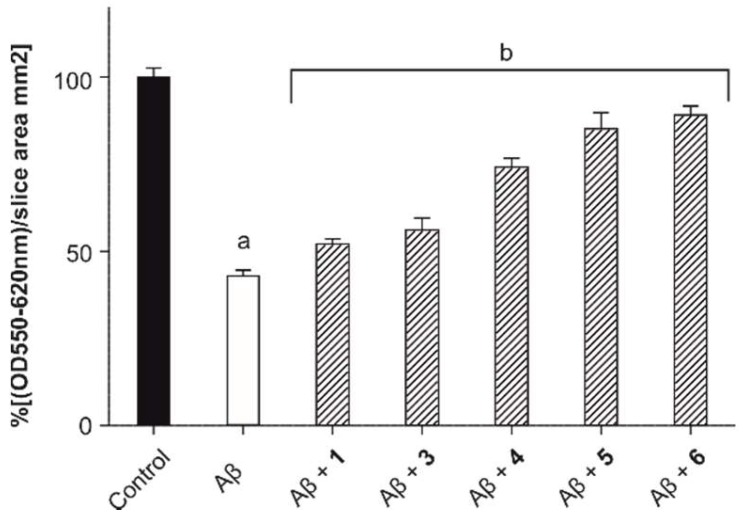
Results of the ex vivo tissue viability test after treatment with Aβ oligomers in 10 µM concentration alone (white column) and in the presence of compounds **1**, **3**–**6** in a 1:1 molar ratio. Untreated slices were used as a control (black column). Data are presented as means ± SD, N = 4. (a, *p* < 0.05, compared to the control and b, *p* < 0.05, compared to the amyloid treated slices).

We have applied a rapid, reliable assay, which utilizes acute hippocampal slices for MTT measurements to test the short-term effect of toxic Aβ oligomers on the viability of the surviving cells in the tissue [38]. Acute hippocampal brain slices can be maintained ex vivo only for a few hours, thus excluding the possibility of studying the long-term effects of neuroprotective compounds. Still, the combination of oxygen-glucose deprivation and treatment with Aβ oligomers makes this assay applicable on a short-term basis, which is otherwise impossible with convenient in vitro methods [38]. The Aβ treatment combined with this short and mild oxygen-glucose deprivation provided a relevant ex vivo model for the aging brain and a standard MTT assay was used for the quantification of toxicity (Table S2). Treatment with Aβ oligomers in 10 µM concentration caused a significant decrease in tissue viability after 4 h (Figure 5, white column, 43.2 ± 1.6% of control). To test the neuroprotective effects of the compounds, slices were treated with Aβ in the presence of equimolar amounts of **1**, **3**–**6**, respectively. In accordance with ITC experiments, MTT tissue viability tests showed a good correlation between the number of arms and biological activity. An increase in the valence of conjugates up to four resulted in an enhanced protection against the Aβ-induced toxicity, but this effect plateaued at the compound **5** and the octavalent conjugate (**6**) did not possess a significantly increased protection against Aβ (89.3 ± 2.7% of control) in comparison with the tetravalent one (5, 85.5 ± 4.8% of control), therefore compound **6** was eliminated from further studies. These outcomes together with the ITC results indicate that, in our case, the biological activity of the compounds could be predictable from binding data.

**Figure 6:**
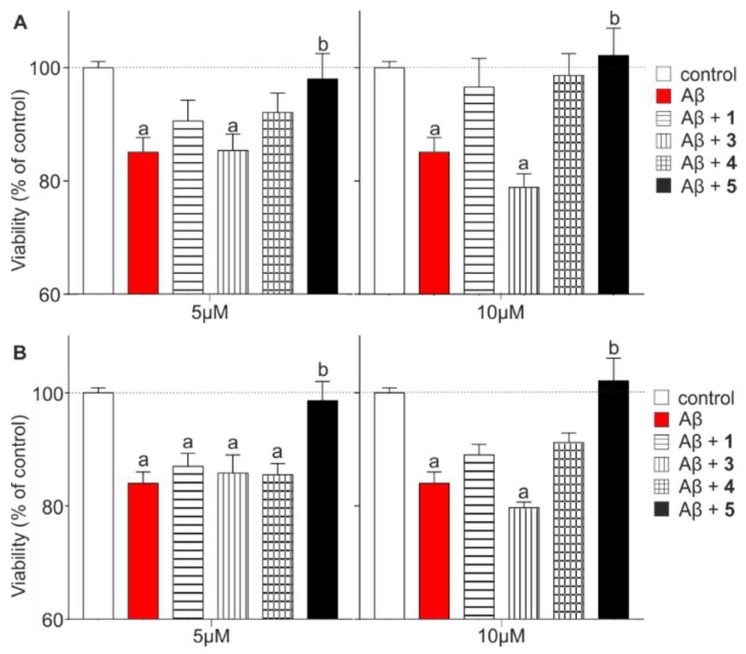
Cell viability readouts based on impedance analysis after treatments at different time points: (**A**) 24 h (**B**) 48 h. Viability of the treated cells was compared to the untreated SHSY-5Y neuroblastoma cells (control group). Aβ oligomer was used in 10 µM concentration in each case: alone (red column) and in the presence of 5 and 10 µM compound **1**, **3**–**5**, respectively. (Statistics: Two-way Anova, mean ± SEM, Bonferroni post-test; a: *p* < 0.05, compared to control, b: *p* < 0.05, compared to Aβ).

Real-time cell impedance measurement assay [39] (xCELLigence) was utilized to test the effects of Aβ and the protective effect of the compounds **1**, **3**–**5** on SH-SY5Y neuroblastoma cells. Cells were treated with Aβ oligomers in the concentration of 10 µM either alone or together with compounds **1**, **3**–**5**, which were applied in two different concentrations (5 or 10 μM). The cell index was monitored continuously for 48 h. Figure 6 represents the viability readouts after 24 h and 48 h (Table S3). Aβ caused a significant reduction in the viability after 24 h (red column, 85.1 ± 2.6% of control) reflecting the effects of an elongated treatment. Compounds **1** and **4** could temporarily increase cell viability (CV) in both concentrations after 24 h (Figure 6A; CV_(**1**; 5 μM)_ = 90.5 ± 3.7%; CV_(**1**; 10 μM)_ = 96.6 ± 5.1%; CV_(**4**; 5 μM)_ = 92.1 ± 3.4%; CV_(**4**; 10 μM)_ = 98.7 ± 3.8%), but this trend disappeared after 48 h (Figure 6B). Interestingly, the viability of cells treated with 5 μM of **3** did not show any change after 24 (CV = 85.4 ± 2.9%) or 48 h (CV = 85.8 ± 3.2%) compared with the Aβ treated cells. Moreover, a higher concentration of **3** further decreased the cell viability (CV_(**3**; 10 μM; 48 h)_ = 79.7 ± 1.0%). Protective compounds **1**, **4** and **5** reduced the toxic effect of Aβ in a concentration-dependent manner at both time points, amongst which the tetravalent conjugate **5** was the most efficient against Aβ-caused toxicity. In this case, cell viability reached the control level already after 24 h at the 5 μM concentration (CV = 98.0 ± 4.5%).

Unlike the MTT tissue viability test, in this experimental setup only the tetravalent foldamer-dendrimer conjugate **5** showed a significant protective effect against Aβ. Differences in the outcomes of the experiments are possibly due to the exposition time and the fact that in a complex system such as an ex vivo tissue (brain slice), neuronal connections and the glial environment remain at least partially intact and Aβ might be able to target different cell types resulting in a multifactorial toxicity, whereas in a monocultural cell model, these multiple effects are missing because of the lower degree of complexity. Therefore, neuronal cell malfunctions leading to viability changes are not fully comparable in these two systems [40,41].

## 3. Materials and Methods

### 3.1. Materials

All Fmoc protected α-amino acids, triisopropylsilane (TIS), Rink Amide AM resin were purchased from Iris Biotech GmbH (Marktredwitz, Germany), β-amino acids, 1, 8-Diazabicyclo [5.4.0] undec-7-ene (DBU), 1-[Bis(dimethylamino)methylene]-1H-1,2,3-triazolo[4,5-b]-pyridinium-3-oxid hexafluorophosphate (HATU) were purchased from GL Biochem (Shanghai, China). Fmoc-(*1S*,*2S*)-ACHC was purchased from PolyPeptide Group (Torrance, CA, USA), Tentagel R RAM resin was purchased from RAPP Polymer GmbH (Tuebingen, Germany). Solvents were purchased from VWR (Radnor, PA, USA). SH-SY5Y human neuroblastoma cells were purchased from Culture Collection (Public Health England, Salisbury, UK; Lot No.: 11C016)

### 3.2. Synthesis

#### 3.2.1. General Methods for the Synthesis of α-Peptidic Scaffolds and β-Peptidic Recognition Segments

Peptides were synthesized manually by SPPS, according to the Fmoc/tBu strategy using Tentagel R RAM resin (capacity: 0.19 mmol g^−1^) and Rink Amide AM resin (0.30 mmol g^−1^). The Fmoc-protecting groups were removed by using 2% piperidine and 2% DBU in *N,N*-dimethylformamide (DMF) (5 + 15 min). Washing procedures were carried out with DMF, dichloromethane (DCM) and methanol. Peptide chain elongation was done by activating three-fold excess of N-Fmoc protected amino acids with HATU/*N,N*-diisopropylethylamine (DIEA) in DMF for 3 h. The peptides were cleaved from the resin with a mixture of trifluoroacetic acid (TFA)/H_2_O/1,4-dithiotreitol (DTT)/TIS (90:5:2.5:2.5) at room temperature for 3 h. TFA was removed in vacuum, and the peptide was precipitated in dried diethyl ether. The resulting free peptide precipitate was filtered off, dissolved in 10% aqueous acetic acid or in the mixture of acetonitrile/H_2_O, and lyophilized. The details of the purification can be found in the Supplementary Information (Table S1).

#### 3.2.2. Synthesis of Compounds **2a**–**2i** and **7**

Different β-peptidic recognition segments listed in Figure 2 and tetra-maleimido-functionalized oligo-l-lysine-dendron scaffolds were synthesized individually by SPPS as described above. Purified lysine-dendron template was dissolved in 50 mM phosphate buffer (pH = 7.0) and added dropwise to the solution of 5.2 eq of different β-peptidic recognition segments in the same buffer under constant stirring. The reaction mixtures were stirred overnight at room temperature. The details of the purification are given in the Supplementary Information (Table S1).

#### 3.2.3. Synthesis of Compound **3**

1,4-Di(maleimido)butane (Sigma-Aldrich, St. Louis, MO, USA) was dissolved in 50 mM phosphate buffer (pH = 7.0) and added dropwise to the solution of 2.6 eq of **1** (Figure 1) dissolved in the same buffer under constant stirring. The reaction was stirred overnight at room temperature. The details of the purification are documented in the Supplementary Information (Table S1).

#### 3.2.4. Synthesis of Compound **4**

Tris(2-aminoethyl) amine (Sigma-Aldrich, St. Louis, MO, USA) and 9 eq of 5-(tert-butoxycarbonylamino)-pentanoic acid/*N,N’*-dicyclohexylcarbodiimide (DCC)/1-hydroxy-7-azabenzotriazole (HOAt) were dissolved in DMF and the mixture was stirred overnight at room temperature, diluted with water and lyophilized. Cleavage of tert-butoxycarbonyl (Boc) protecting group was carried out with the mixture of TFA/DCM (1:1) at room temperature for 30 min. The reaction mixture was diluted with the mixture of 0.1% TFA/H_2_O, lyophilized and purified by RP-HPLC. The purified peptide was dissolved in DMF and it was added dropwise to the mixture of 9 eq of 3-maleimidopropionic acid (MPA)/DCC/HOAt in DMF. The reaction mixture was stirred for 4 h at room temperature, diluted with water and lyophilized. The purified functionalized scaffold was dissolved in 50 mM phosphate buffer (pH = 7.0) and added dropwise to the solution of the 3.9 eq of **1** dissolved in the same buffer under constant stirring. The reaction was stirred overnight at room temperature. The details of the purification are given in the Supplementary Information (Table S1).

#### 3.2.5. Synthesis of Compound **5**

Detailed synthesis of compound **5** was reported previously in reference [35].

#### 3.2.6. Synthesis of Compound **6**

Four hundred microliters of 20 wt% methanol solution of polyamidoamine dendrimer generation 1, ethylenediamine core (G1-PAMAM, 0.23 mmol, Sigma-Aldrich, St. Louis, MO, USA) was lyophilized for 1 h to remove methanol. The resulting product was dissolved in DMF and added dropwise to the mixture of 24 eq (5.52 mmol) of MPA/ DCC/ HOAt dissolved in DMF. The mixture was stirred for 4 h at room temperature, diluted with water and lyophilized to dryness. The purified and lyophilized octa-maleimido-functionalized G1-PAMAM was dissolved in 50 mM phosphate buffer (pH = 7.0) and added dropwise to the solution of 10.4 eq of **1** dissolved in the same buffer under constant stirring. The reaction was stirred overnight at room temperature. The details of the purification are documented in the Supplementary Information (Table S1).

#### 3.2.7. Synthesis of Compound **8**

Ac-KGKGKGK-NH_2_ peptide sequence was synthesized manually by SPPS as described above. The purified peptide was dissolved in DMF and was added dropwise to a pre-activated mixture of 12 eq of MPA/DCC/HOAt in DMF. The reaction mixture was stirred for 4 h at room temperature, diluted with water and lyophilized. Ac-K(MPA)GK(MPA)GK(MPA)GK(MPA)-NH_2_ was dissolved in 50 mM phosphate buffer (pH = 7.0) and added dropwise to the solution of 5.2 eq of **1** dissolved in the same buffer under constant stirring. The reaction was stirred overnight at room temperature. The details of the purification are presented in the Supplementary Information (Table S1).

#### 3.2.8. Synthesis of Compound **9**

Compound **1** was elongated with Fmoc-PEG2-Suc-OH (Eurogentec, Seraing, Belgium) and (*1S*,*2S*)-ACHC-β^3^homoArg-(*1S*,*2S*)-ACHC-(*1S*,*2S*)-ACHC-β^3^homoAsp-(*1S*,*2S*)-ACHC-NH_2_ was built up on the *N*-terminal end of the sequence. The peptide was cleaved from the resin and purified. Oxidative coupling of thiols to disulfides was carried out with freshly prepared 1% (*w*/*v*) I_2_ solution in acetone, which was added dropwise to the solution of thiol functionalized peptide in H_2_O under constant stirring at room temperature. The completion of the reaction was monitored by analytical HPLC. The details of the purification can be found in the Supplementary Information (Table S1).

### 3.3. Preparation of Aβ Samples

In this work, Ser^26^ depsipeptide *iso*-Aβ (1–42) was used, which was synthesized and purified as described previously [42]. The lyophilized *iso*-Aβ (1–42) was dissolved in MilliQ water to a concentration of 1 mg mL^−1^, sonicated for 3 min and the pH was set to 7.0 to initiate an O to N acyl migration, whereby the native sequence can be readily formed. The sample was incubated at room temperature for 10 min. Then the pH was set to 11 and the sample was kept at room temperature for additional 2 h. After the incubation, the Aβ stock solution was aliquoted and stored at −20 °C until use. To obtain Aβ oligomers, the 1 mg mL^−1^ aliquot was diluted to a final concentration of 50 μM with 26.67 mM PBS and the pH was set to 7.4 with 1 M HCl. The sample was incubated at 37 °C for 3 h. To calculate the accurate concentration of the Aβ solution, the peptide content of the lyophilized *iso*-Aβ (1–42) was determined by amino acid analysis, and it varied typically between 70–80%. Exact peptide concentrations of the stock solution were calculated by taking these data in consideration.

### 3.4. ITC Measurements

Isothermal calorimetric titrations were performed with a Microcal VP-ITC microcalorimeter in pH 7.4 PBS buffer solution. In individual titrations, 10 µL portions of the ligand containing solution were repeatedly injected from the computer-controlled 300-µL microsyringe at intervals of 300 s into the Aβ oligomer solution prepared in the same buffer as the ligand. All measurements were carried out at 285 K. The Aβ concentration in the cell was 100 µM and the total ligand concentration was 250 µM in the syringe and the titration was stopped when the precipitation of the Aβ aggregates became excessive. Control experiments were performed by injecting the ligand into a cell containing buffer with no target, and the heats of dilution were subtracted from those measured in the presence of Aβ. The experimental data were fitted to the two independent site binding models by using a nonlinear least-squares procedure, with ΔHb, ΔHb’, Kd, Kd’ (association constants), n and n’ (number of binding sites for monomer), as adjustable parameters.

### 3.5. Ex Vivo Testing Aβ (1–42) Toxicity on Acute Hippocampal Slices

Toxicity measurements were carried out using freshly prepared slices (N = 2 × 10 for one case) of the brains of 10 ± 1-week-old Wistar rats (4 animals, thickness of the brain slices: 400 µm). After the area determination, slices were conditioned in a carboxygenated (95/5%: O_2_/CO_2_) preparation solution for 30 min at room temperature. Then the tissues were left to rest at room temperature in glucose- and carboxygen-free solution for 1 h. After the oxygen-glucose deprivation (OGD), slices were immediately treated with freshly prepared oligomeric Aβ peptide in a final concentration of 10 μM alone and in the presence of protective compounds, each one in a concentration of 10 μM. After the treatment, brain slice viability was measured using a specific MTT assay. For details of the OGD protocol and MTT measurement see reference [38]. Experiments were performed in accordance with the European Communities Council Directive of 22 September 2010 (2010/63/EU on the protection of animals used for scientific purposes). The animal protocols applied in this study were approved by the National Institute of Health, and by the University of Szeged; permission number: I-02442/001/2006.

### 3.6. Impedance Based Measurement of Aβ Toxicity on Neuroblastoma Cells

Differentiated SH-SY5Y cells were grown in Dulbecco’s Modified Eagle’s Medium (DMEM) and F12-Ham’s culture medium at a 1:1 ratio supplemented with 10% (*v*/*v*) fetal bovine serum (FBS), 1% (*v/v*) l-glutamine, and 50 µg/mL gentamicin. Cells were cultured in a humidified, 5% (*v/v*) CO_2_, 37 °C incubator. Plates with integrated gold electrodes (E-plate, 96-well format, RTCA-SP instrument; ACEA Biosciences, San Diego, CA, USA) were coated with 5% rat tail collagen-PBS solution. For background measurements, 50 µL cell culture medium was added to the wells, then cells were seeded at a density of 6 × 10^3^/well. Cells were cultured for 5–7 days and monitored every 15 min until the end of experiments. At the beginning of plateau phase of growth, cells were treated with Aβ peptide alone or in the presence of compounds. Cells treated with vehicle served as a control group. The effects of the treatments were followed for 48 h (N = 4–8). Impedance measurement is label-free, real time, noninvasive, and correlates linearly with adherence, growth, number and viability of cells [43]. The cell index at each time point was defined as (R_n_ − R_b_)/15, where R_n_ is the cell-electrode impedance of the well when it contains cells and R_b_ is the background impedance of the well with the medium alone. The resulting data was analyzed using real-time cell analyzer software (RTCA, Roche, Basel, Switzerland) and exported to Excel.

### 3.7. Statistical Analysis

Data are presented as means ± standard deviation (SD) in each case, expecting impedance-based cell viability test, where data are presented as means ± standard error of mean (SEM). After ANOVA, Bonferroni post hoc test was used for statistical evaluation using GraphPad Prism version 5.03 (GraphPad Software, La Jolla, CA, USA) for Windows software. Differences were considered statistically significant at *p* < 0.05.

## 4. Conclusion

Inhibiting protein-protein interactions is among the greatest challenges in drug discovery, and β-peptide foldamers can be prominent drug candidates as alternatives to therapeutic proteins in this regard. Operating with multiple molecular recognition elements is an excellent tool in drug research [44,45] including Alzheimer’s disease research [46,47]. Although the direct neutralization of toxic Aβ species could be a potential therapeutic strategy in AD [48], targeting the solvent-exposed potential binding sites of the partially disordered Aβ oligomers with small synthetic molecules is a real challenge. The combination of multivalent design with foldamer methodology can provide an opportunity to inhibit the effect of a protein-like species with a complex structure such as Aβ oligomers. In this study two strategies were followed in parallel for the structure optimization of the previously published tetravalent foldamer-conjugate [35]: Altering the side chain chemistry of the foldamer fragment and changing the valence and the topology of the conjugate. Optimized multivalent foldamer constructions show promising biological activity against toxicity of Aβ. Based on the binding and viability studies, substantial structural features of the Aβ recognizing oligomers can be defined which allow us to understand the interaction and can help us target neurotoxic Aβ oligomers more efficiently in future applications.

## Figures and Tables

**Table 1:** Thermodynamic parameters as resulted from ITC analysis for compounds **2a**–**2i** and K_D_ values from quantitative evaluation of ELISA experiments.

Compound	K_D ITC_ (nM^−1^)	ΔG (kcal M^−1^)	ΔH (kcal M^−1^)	−TΔS (kcal M^−1^)	K_D ELISA_ (nM)
**2a**	27.63 ± 7.74 ^1^	−9.97	51.33	61.30	0.95 ± 0.06
	239.62 ± 68.67 ^2^	−8.73	0.31	9.04	
**2b**	53.20 ± 38.70 ^1^	−9.59	6.57	16.16	5.36 ± 1.42
	373.40 ± 104.33 ^2^	−8.78	0.64	9.42	
**2c**	2.53 ± 1.81 ^1^	−11.34	1.96	13.30	1.92 ± 0.21
	175.20 ± 46.41 ^2^	−8.91	0.76	9.67	
**2d**	ND	ND	ND	ND	7.70 ± 3.06
**2e**	ND	ND	ND	ND	2.71 ± 0.21
**2f**	19.30 ± 9.40 ^1^	−10.17	2.48	12.66	1.79 ± 0.09
	816.40 ± 466.90 ^2^	−8.03	0.27	8.30	
**2g**	34.10 ± 10.00 ^1^	−9.85	4.88	14.73	1.34 ± 0 04
	652.30 ± 158.50 ^2^	−8.16	0.37	8.53	
**2h**	8.61 ± 5.64 ^1^	−10.64	7.39	18.02	1.35 ± 0.08
	78.99 ± 45.92 ^2^	−9.37	0.35	9.72	
**2i**	ND	ND	ND	ND	1.59 ± 0 08

ND = not determined. ^1^ K_D_ belongs to the first binding stage. ^2^ K_D_ belongs to the second binding stage.

**Table 2:** Thermodynamic parameters from ITC analysis of compounds **1**, **3**–**9**.

Compound	K_D_ (nM^−1^)	ΔG (kcal M^−1^)	ΔH (kcal M^−1^)	−TΔS (kcal M^−1^)
**1**	2376.1 ± 214.4 *	−7.42 *	0.36 *	7.77 *
**3**	721.4 ± 120.1 *	−8.10 *	1.14 *	9.24 *
**4**	18.5 ± 13.9 ^1^	−10.20	2.16	12.36
	155.0 ± 130.9 ^2^	−8.98	0.28	9.26
**5**	6.9 ± 1.4 ^1^	−10.38	10.01	20.39
	281.1 ± 38.7 ^2^	−8.25	2.55	10.80
**6**	69.0 ± 12.0 ^1^	−9.45	49.4	58.85
	193.4 ± 30.5 ^2^	−8.88	0.31	9.17
**7**	16.2 ± 8.9 ^1^	−10.27	9.29	19.56
	127.6 ± 56.7 ^2^	−9.09	0.23	9.32
**8**	4.1 ± 2.5 ^1^	−11.07	2.31	13.38
	374.0 ± 102.0 ^2^	−8.48	0.51	8.99
**9**	35.0 ± 19.7 ^1^	−9.83	6.01	15.84
	372.1 ± 212.8 ^2^	−8.48	1.18	9.66

* data are taken from reference [35]. ^1^ K_D_ belongs to the first binding stage. ^2^ K_D_ belongs to the second binding stage.

## Supplementary Materials

The following are available online. Figure S1: ITC titration data for **2a**–**2d** and **2f**–**2h**, Figure S2: The results of the ELISA experiment of **2a**–**2i**, Figure S3: ITC enthalpogram for the titration of Aβ with different conjugate (**4**–**9**), Table S1: Details of the purification of compounds, Table S2: Results of the ex vivo tissue viability test, Table S3: Cell viability readouts based on impedance analysis.

## References

[1] Eisele Y.S., Monteiro C., Fearns C., Encalada S.E., Wiseman R.L., Powers E.T., Kelly J.W. (2015). Targeting protein aggregation for the treatment of degenerative diseases. Nat. Rev. Drug Discov..

[2] Aguzzi A., O’connor T. (2010). Protein aggregation diseases: Pathogenicity and therapeutic perspectives. Nat. Rev. Drug Discov..

[3] Hardy J., Selkoe D.J. (2002). The amyloid hypothesis of Alzheimer’s disease: Progress and problems on the road to therapeutics. Science.

[4] Selkoe D.J. (2002). Alzheimer’s disease is a synaptic failure. Science.

[5] Haass C., Selkoe D.J. (2007). Soluble protein oligomers in neurodegeneration: Lessons from the Alzheimer’s amyloid [beta]-peptide. Nat. Rev. Mol. Cell Biol..

[6] Kayed R., Head E., Thompson J.L., McIntire T.M., Milton S.C., Cotman C.W., Glabe C.G. (2003). Common structure of soluble amyloid oligomers implies common mechanism of pathogenesis. Science.

[7] Selkoe D.J., Hardy J. (2016). The amyloid hypothesis of Alzheimer’s disease at 25 years. EMBO Mol. Med..

[8] Yan R., Vassar R. (2014). Targeting the β secretase BACE1 for Alzheimer’s disease therapy. Lancet Neurol..

[9] Golde T.E., Koo E.H., Felsenstein K.M., Osborne B.A., Miele L. (2013). γ-Secretase inhibitors and modulators. BBA-Biomembranes.

[10] Nishiyama Y., Taguchi H., Hara M., Planque S.A., Mitsuda Y., Paul S. (2014). Metal-dependent amyloid β-degrading catalytic antibody construct. J. Biotechnol..

[11] DeMattos R.B., Bales K.R., Cummins D.J., Dodart J.C., Paul S.M., Holtzman D.M. (2001). Peripheral anti-Aβ antibody alters CNS and plasma Aβ clearance and decreases brain Aβ burden in a mouse model of Alzheimer’s disease. Proc. Natl. Acad. Sci. USA.

[12] Nicoll J.A., Wilkinson D., Holmes C., Steart P., Markham H., Weller R.O. (2003). Neuropathology of human Alzheimer disease after immunization with amyloid-β peptide: A case report. Nat. Med..

[13] Nicoll J.A., Barton E., Boche D., Neal J.W., Ferrer I., Thompson P., Vlachouli C., Wilkinson D., Bayer A., Games D. (2006). Aβ species removal after Aβ42 immunization. J. Neuropath. Exp. Neur..

[14] Hawkes C.A., Ng V., McLaurin J. (2009). Small molecule inhibitors of Aβ-aggregation and neurotoxicity. Drug Dev. Res..

[15] Bruinsma I.B., Karawajczyk A., Schaftenaar G., de Waal R.M., Verbeek M.M., van Delft F.L. (2011). A rational design to create hybrid β-sheet breaker peptides to inhibit aggregation and toxicity of amyloid-β. MedChemComm.

[16] Guisasola E.E.B., Andujar S.A., Hubin E., Broersen K., Kraan I.M., Méndez L., Delpiccolo C.M.L., Masman M.F., Rodríguez A.M., Enriz R.D. (2015). New mimetic peptides inhibitors of Aβ aggregation. Molecular guidance for rational drug design. Eur. J. Med. Chem..

[17] Paul A., Nadimpally K.C., Mondal T., Thalluri K., Mandal B. (2015). Inhibition of Alzheimer’s amyloid-β peptide aggregation and its disruption by a conformationally restricted α/β hybrid peptide. Chem. Commun..

[18] Ross C.A., Poirier M.A. (2004). Protein aggregation and neurodegenerative disease. Nat. Med..

[19] Arkin M.R., Tang Y., Wells J.A. (2014). Small-molecule inhibitors of protein-protein interactions: Progressing toward the reality. Chem. Biol..

[20] Scott D.E., Bayly A.R., Abell C., Skidmore J. (2016). Small molecules, big targets: Drug discovery faces the protein–protein interaction challenge. Nat. Rev. Drug Discov..

[21] Arkin M.R., Wells J.A. (2004). Small-molecule inhibitors of protein-protein interactions: Progressing towards the dream. Nat. Rev. Drug Discov..

[22] Jones S., Thornton J.M. (1997). Analysis of protein-protein interaction sites using surface patches. J. Mol. Biol..

[23] Bogan A.A., Thorn K.S. (1998). Anatomy of hot spots in protein interfaces. J. Mol. Biol..

[24] Sela-Culang I., Kunik V., Ofran Y. (2013). The structural basis of antibody-antigen recognition. Front. Immunol..

[25] Scott A.M., Wolchok J.D., Old L.J. (2012). Antibody therapy of cancer. Nat. Rev. Cancer.

[26] Weiner L.M., Surana R., Wang S. (2010). Monoclonal antibodies: Versatile platforms for cancer immunotherapy. Nat. Rev. Immunol..

[27] Werner H.M., Horne W.S. (2015). Folding and function in α/β-peptides: Targets and therapeutic applications. Cur.r Opin. Chem. Biol..

[28] Guichard G., Huc I. (2011). Synthetic foldamers. Chem. Commun..

[29] Goodman C.M., Choi S., Shandler S., DeGrado W.F. (2007). Foldamers as versatile frameworks for the design and evolution of function. Nat. Chem. Biol..

[30] Martinek T.A., Fülöp F. (2012). Peptidic foldamers: Ramping up diversity. Chem. Soc. Rev..

[31] Gademann K., Ernst M., Hoyer D., Seebach D. (1999). Synthesis and Biological Evaluation of a Cyclo-β-tetrapeptide as a Somatostatin Analogue. Angew. Chem. Int. Ed..

[32] Johnson L.M., Barrick S., Hager M.V., McFedries A., Homan E.A., Rabaglia M.E., Keller M.P., Attie A.D., Saghatelian A., Bisello A. (2014). A potent α/β-peptide analogue of GLP-1 with prolonged action in vivo. J. Am. Chem. Soc..

[33] Horne W.S., Boersma M.D., Windsor M.A., Gellman S.H. (2008). Sequence-Based Design of α/β-Peptide Foldamers That Mimic BH3 Domains. Angew. Chem. Int. Ed..

[34] Cabrele C., Martinek T.A., Reiser O., Berlicki Ł. (2014). Peptides containing β-amino acid patterns: Challenges and successes in medicinal chemistry. J. Med. Chem..

[35] Fülöp L., Mándity I.M., Juhász G., Szegedi V., Hetényi A., Wéber E., Bozsó Z., Simon D., Benkő M., Király Z. (2012). A foldamer-dendrimer conjugate neutralizes synaptotoxic β-amyloid oligomers. PLoS ONE.

[36] Olajos G., Bartus E., Schuster I., Lautner G., Gyurcsányi R.E., Szögi T., Fülöp L., Martinek T.A. (2017). Multivalent foldamer-based affinity assay for selective recognition of Aβ oligomers. Anal. Chim. Acta.

[37] Li L., Vorobyov I., Allen T.W. (2013). The different interactions of lysine and arginine side chains with lipid membranes. J. Phys. Chem. B.

[38] Mozes E., Hunya A., Posa A., Penke B., Datki Z. (2012). A novel method for the rapid determination of beta-amyloid toxicity on acute hippocampal slices using MTT and LDH assays. Brain Res. Bull..

[39] Ke N., Wang X., Xu X., Abassi Y.A. (2011). The xCELLigence system for real-time and label-free monitoring of cell viability. Mamm. Cell Viability.

[40] Hettiarachchi N., Dallas M., Al-Owais M., Griffiths H., Hooper N., Scragg J., Boyle J., Peers C. (2014). Heme oxygenase-1 protects against Alzheimer’s amyloid-β1-42-induced toxicity via carbon monoxide production. Cell Death Dis..

[41] LeBlanc A.C. (2005). The role of apoptotic pathways in Alzheimer’s disease neurodegeneration and cell death. Curr. Alzheimer Res..

[42] Bozso Z., Penke B., Simon D., Laczkó I., Juhász G., Szegedi V., Kasza A., Soós K., Hetényi A., Wéber E. (2010). Controlled in situ preparation of Aβ (1–42) oligomers from the isopeptide “iso-Aβ (1–42)”, physicochemical and biological characterization. Peptides.

[43] Kiss L., Walter F.R., Bocsik A., Veszelka S., Ózsvári B., Puskás L.G., Szabó-Révész P., Deli M.A. (2013). Kinetic analysis of the toxicity of pharmaceutical excipients Cremophor EL and RH40 on endothelial and epithelial cells. J. Pharm. Sci..

[44] Boas U., Heegaard P.M. (2004). Dendrimers in drug research. Chem. Soc. Rev..

[45] Lee C.C., MacKay J.A., Fréchet J.M., Szoka F.C. (2005). Designing dendrimers for biological applications. Nat. Biotechnol..

[46] Chafekar S.M., Malda H., Merkx M., Meijer E.W., Viertl D., Lashuel H.A., Baas F., Scheper W. (2007). Branched KLVFF Tetramers Strongly Potentiate Inhibition of β-Amyloid Aggregation. ChemBioChem.

[47] Kim Y., Lee J.H., Ryu J., Kim D.J. (2009). Multivalent & multifunctional ligands to β-amyloid. Curr. Pharm. Design.

[48] Wisniewski T., Goñi F. (2015). Immunotherapeutic approaches for Alzheimer’s disease. Neuron.

